# Cell‐free DNA from ascites identifies clinically relevant variants and tumour evolution in patients with advanced ovarian cancer

**DOI:** 10.1002/1878-0261.13710

**Published:** 2024-08-08

**Authors:** Bonnita Werner, Elyse Powell, Jennifer Duggan, Marilisa Cortesi, Yeh Chen Lee, Vivek Arora, Ramanand Athavale, Michael Dean, Kristina Warton, Caroline E. Ford

**Affiliations:** ^1^ Gynaecological Cancer Research Group, School of Clinical Medicine, Faculty of Medicine and Health University of New South Wales Sydney Australia; ^2^ Gynaecological Oncology Department Royal Hospital for Women Sydney Australia; ^3^ Laboratory of Cellular and Molecular Engineering, Department of Electrical, Electronic and Information Engineering Alma Mater Studiorum‐University of Bologna Italy; ^4^ School of Clinical Medicine, Faculty of Medicine and Health University of New South Wales Sydney Australia; ^5^ Prince of Wales Private Hospital Sydney Australia; ^6^ Laboratory of Translational Genomics, Division of Cancer Epidemiology and Genetics National Cancer Institute Rockville MD USA

**Keywords:** ascites, biomarkers, cell‐free DNA, homologous recombination deficiency, ovarian cancer, precision medicine

## Abstract

The emergence of targeted therapies has transformed ovarian cancer treatment. However, biomarker profiling for precision medicine is limited by access to quality, tumour‐enriched tissue samples. The use of cell‐free DNA (cfDNA) in ascites presents a potential solution to this challenge. In this study, next‐generation sequencing was performed on ascites‐derived cfDNA samples (26 samples from 15 human participants with ovarian cancer), with matched DNA from ascites‐derived tumour cells (*n* = 5) and archived formalin‐fixed paraffin‐embedded (FFPE) tissue (*n* = 5). Similar tumour purity and variant detection were achieved with cfDNA compared to FFPE and ascites cell DNA. Analysis of large‐scale genomic alterations, loss of heterozygosity and tumour mutation burden identified six cases of high genomic instability (including four with pathogenic *BRCA1* and *BRCA2* mutations). Copy number profiles and subclone prevalence changed between sequential ascites samples, particularly in a case where deletions and chromothripsis in Chr17p13.1 and Chr8q resulted in changes in clinically relevant *TP53* and *MYC* variants over time. Ascites cfDNA identified clinically actionable information, concordant to tissue biopsies, enabling opportunistic molecular profiling. This advocates for analysis of ascites cfDNA *in lieu* of accessing tumour tissue via biopsy.

## Graphical abstract

The analysis of DNA extracted from ascites enables an opportunistic approach to personalising treatment for ovarian cancer. This article demonstrates that cell‐free DNA in ascites can provide the most accurate and insightful information at key timepoints throughout disease history. Analysis of ascites cell‐free DNA can direct clinical action, such as use of PARP inhibitors, prior to surgery or after tumour evolution.

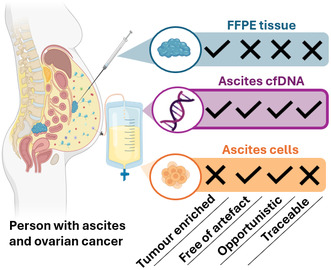

**Abbreviations**
cfDNA: cell‐free DNA
DNA: deoxyribonucleic acid
FFPE: formalin‐fixed paraffin‐embedded
HRD: homologous recombination deficient
LGA: large‐scale genome alteration
LOH: loss of heterozygosity
ONT: Oxford Nanopore Technologies
sphDNA: ascites cell spheroid DNA
TMB: tumour mutation burden
VAF: variant allele frequency

## 1 Introduction

**Fig. 1:**
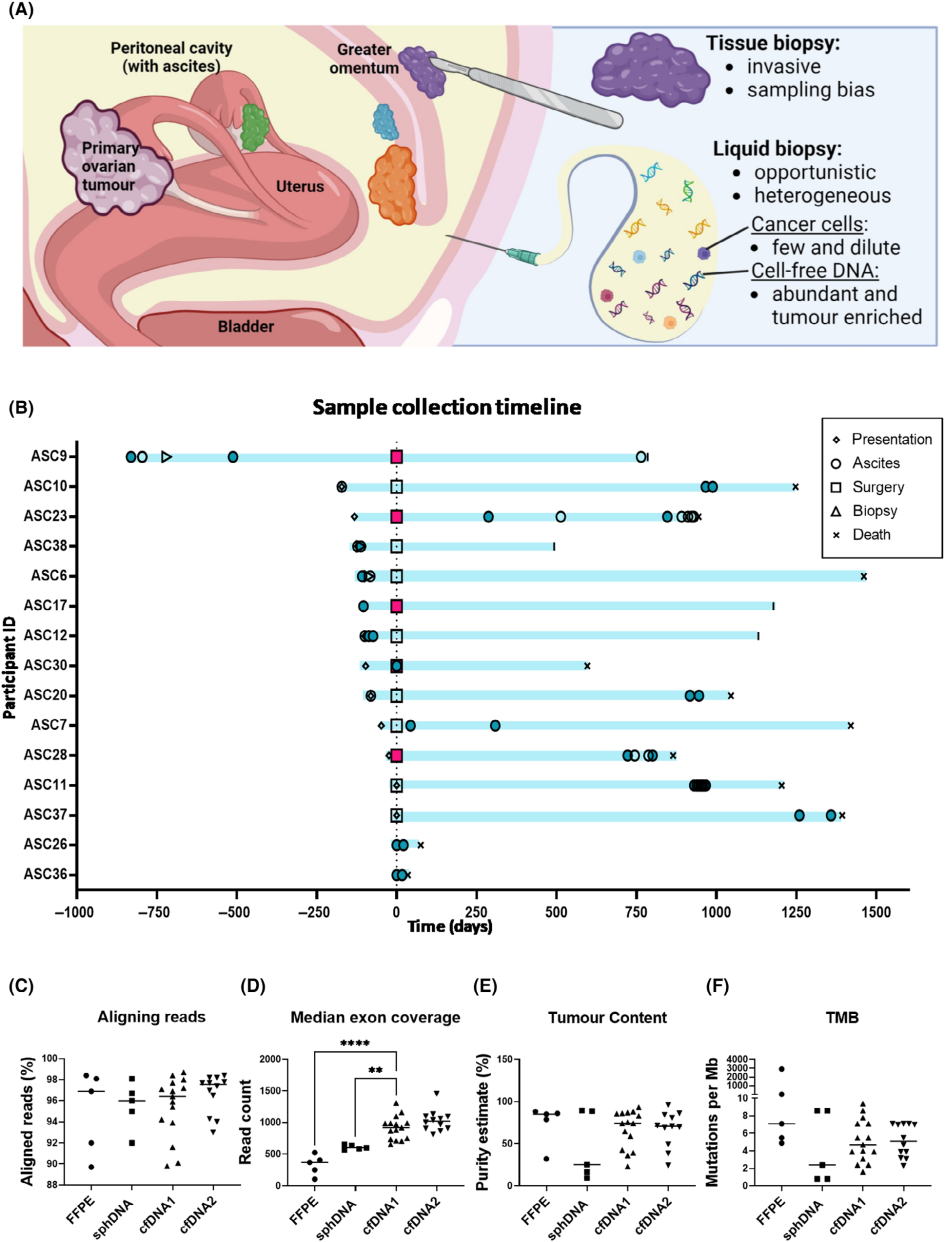
Sample collection overview and quality metrics. (A) Advantages and limitations of tissue and liquid biopsies. Made using Biorender.com. (B) Timeline of sample collection events with samples included in study as solid shapes. (C) Percentage of reads successfully aligning to GRCh37, (D) median read depth over targeted exons, (E) tumour purity estimated from deleterious TP53 VAF and (F) tumour mutation burden (TMB) determined by TSO500 local app v2.2, compared by Dunnett's multiple comparisons test (though serial cfDNA samples are visualised, only the first sample is included in analysis). cfDNA, cell‐free DNA; FFPE, DNA from formalin‐fixed paraffin‐embedded tissue; Mb, megabase; sphDNA, DNA from ascites‐derived cell spheroids; VAF, variant allele frequency; **P < 0.01; ****P < 0.0001.

The rise of targeted agents has seen a pivotal shift in ovarian cancer treatment [1]. Consequently, individualised biomarker profiling is now standard clinical practice [2]. Biomarker identification is reliant on accessible, tumour‐enriched DNA sources, which can be a challenge, particularly in circumstances where excisional or core biopsies are contraindicated or intolerable to the patient [3, 4]. Ascites is a common feature of advanced ovarian cancer and may provide access to informative, heterogenous tumour tissue *in lieu* of a tissue biopsy, avoiding the need for additional invasive procedures (Fig. 1A).

Ascites is present at the time of diagnosis in over 90% of stage III and IV ovarian cancers, where most diagnoses are made [5], and is drained to relieve the debilitating symptoms it causes [6]. The incidentally collected ascites samples are often used as surrogate biopsies for ovarian cancer, to aid diagnosis [7]. However, approximately 20% of these specimens are cytologically classified as non‐malignant due to absence of cancer cells, hindering performance of molecular tumour analysis [8, 9]. Tumour enrichment of ascites cells can be accomplished by collecting only cell spheroids (the metastatic drivers of ovarian cancer) [10]; however, we have previously shown cell‐free DNA (cfDNA) to improve on spheroids as a source of concentrated and abundant tumour DNA [11]. This is in alignment with other reports demonstrating that ascites‐derived cfDNA is tumour enriched [12], even in cases where malignant cells are absent from the fluid [9, 13]. Representative (even unique) mutational profiles have been proven identifiable in cfDNA and homologous recombination deficiency (HRD) scores that align with solid tumours have also been elucidated [14, 15, 16]. Consequently, recommendations have been made to implement cfDNA testing in routine clinical practice. However, limited focus has been placed on verifying unique mutations in cfDNA.

As ascites poses a poor prognosis for primary debulking surgery [17], neoadjuvant chemotherapy is often used [18], delaying access to tumour tissue for molecular profiling, or necessitating invasive and often unsuccessful core biopsies [19, 20]. Tissue collected at interval debulking surgery often performs poorly in sequencing, due to chemotherapy causing the death of tumour cells and increasing immune infiltrate, diluting tumour DNA [21]. Additionally, ascites occurs in most cases of relapse, where further surgery is rarely performed [3]. Relapsed ovarian cancer is often molecularly distinct from its predecessor [22, 23]. Thereby cfDNA in ascites may provide earlier access to an uncompromised tumour sample, as well as unique access to evolved disease. However, of studies on ovarian cancer ascites cfDNA, none have centred on sequential ascites samples for the temporal analysis of ovarian cancer.

In this study, we aimed to understand whether new and useful information could be gained from ascites cfDNA at different time points, with a focus on whether this information is reliable and reproducible. We assess the feasibility of applying targeted next‐generation sequencing to ascites cfDNA to identify actionable biomarkers, and we evaluate concordance with ascites tumour cells, archived formalin‐fixed tumour tissue and clinical reports. We also compare cfDNA from sequential ascites samples to ascertain whether opportune ascites sampling reveals time‐critical changes that may inform personalised disease management.

## 2 Materials and methods

### 2.1 Cohort selection and sample collection

Up to 1 L of ascites was collected into sterile, untreated plastic bottles from patients of the Royal Hospital for Women, Sydney, with confirmed or suspected ovarian cancer, between June, 2019 and March, 2022. Samples were collected with informed written consent during routine paracentesis appointments, with approval from the Prince of Wales Hospital Human Research Ethics Committee (HC19‐001), conforming to the standards set by the Declaration of Helsinki. The 15 person cohort for this study (as part of a larger study [11]) was selected based on meeting any of the ordered priorities when this study began: multiple ascites samples collected; clinical sequencing report available identifying deleterious *BRCA1/2* variants (germline or somatic); time‐matched tissue sample available; clinical sequencing report available identifying deleterious somatic variants (Fig. S1). Where available, FFPE tumour biopsy samples were retrieved through the Health Systems Alliance Biobank, UNSW.

### 2.2 Sample processing and DNA extraction

Samples were processed within 24 h to fractionate cell‐free fluid and cell spheroid pellets, as previously described [11]. Briefly, fluid was passed through a 40 μm filter, capturing cell spheroids. Filtrate underwent two centrifugations to isolate the ‘cell‐free’ component. cfDNA was extracted from cell‐free ascites fluid using the QIAamp Circulating Nucleic Acid Kit (Qiagen, Hilden, Germany). DNA was extracted from cell spheroid pellets using the All‐In‐One DNA/RNA/Protein Miniprep kit (Bio Basic Inc., Markham, ON, Canada). FFPE tissue was first treated with de‐paraffanisation solution (Qiagen), then DNA was extracted using the NucleoSpin DNA FFPE XS, Microkit for DNA from FFPE (Macherey‐Nagel, Düren, Germany) or the QIAamp DNA FFPE Tissue Kit (Qiagen). Primary samples and extracted DNA were stored at −80 °C.

### 2.3 Next‐generation sequencing

TruSight Oncology 500 (TSO500) libraries (Illumina, San Diego, CA, USA), covering 523 cancer‐related genes, were prepared at the Ramaciotti Centre for Genomics, UNSW. DNA integrity was assessed using the Agilent gDNA ScreenTape or the Cell‐free DNA ScreenTape on the Agilent TapeStation 4200, for spheroid DNA (sphDNA) and cfDNA, respectively. FFPE DNA integrity was tested with the Illumina FFPE QC Kit.

Samples were prepared according to the Illumina TSO500 High Throughput Reference Guide, following the DNA only workflow, with a minimum of 40 ng input, measured by Qubit High Sensitivity DNA Assay (Thermo Fisher Scientific, Waltham, MA, USA). sphDNA and FFPE samples were fragmented using the Covaris E220, then checked on the High Sensitivity D1000 ScreenTape assay. cfDNA samples were not fragmented.

Final libraries were checked on the High Sensitivity D1000 ScreenTape assay and bead‐based normalisation was performed to ensure quality and uniformity prior to pooling. Library pools were prepared following the relevant instructions in the NovaSeq 6000 Denature and Dilute Libraries Guide. Samples were sequenced on the NovaSeq 6000 using either the S1 200‐cycle kit or the SP 300‐cycle kit (XP protocol), following manufacturer instruction.

Oxford Nanopore Technology (ONT) sequencing library for participant ASC23 was created from ~ 30 ng cfDNA (50 fmol sequenced) using the ligation sequencing gDNA kit, SQK‐LSK110 and an R9.4.1 flow cell (ONT, Oxford, UK), with some modifications for cfDNA as recommended by ONT (https://community.nanoporetech.com/extraction_methods/human‐blood‐cfdna).

### 2.4 Data processing and analysis

#### 2.4.1 Small variant profiling

Sequencing data was processed using the TSO500 v2.2 Local Application (TSO500 Local App), aligning to reference genome hg19/GRCh37 [24]. The app reported tumour mutation burden (TMB) and gene‐specific amplifications.

Total variants were counted and samples with total variant count outside of a *Z*‐score (from a mean excluding one majorly outlying sample) of |3| were identified. As we had no germline reference samples, variants were considered likely somatic if they had a minor allele count of < 100 in each of three population databases (< 0.05% GnomAD Exome, < 0.5% GnomAD Genome and < 2% 1000 Genomes). Of these, single and multiple nucleotide polymorphisms were assessed by the Cancer Genome Interpreter [25], using SNPnexus [26], to assess their likelihood of cancer driving capacity. ClinVar, Varsome and COSMIC were used to similarly assess insertions and deletions.

Integrated genome viewer was used to verify VAF and read depth of any variants with non‐concordance across patient‐matched samples. Variants were considered verifiable where reported in > 1 sample per participant, or if unique, where > 0.05 variant allele frequency (VAF) and > 150× read depth. Integrated Genome Viewer was also used to manually search for reversion mutations which restored reading frame, corrected premature stop codon or restored a missense codon in people with pathogenic variants in HRD‐related genes.

Single base substitution signatures of likely somatic variants were identified using SigProfiler tools, SigProfiler Matrix Generator and SigProfiler Extractor, using the computational cluster Katana [27].

#### 2.4.2 Tumour purity

Tumour purity was estimated based on the typical trajectory of HGSOC carcinogenesis, where if a single deleterious *TP53* mutation was present, the competing healthy allele is deleted, conforming to the double‐hit hypothesis [28]. The following formula was used, where *t* is tumour fraction and *v* is the variant allele frequency (VAF) of the deleterious *TP53* variant.

$$t=v/\left(\left(\frac{1-v}{2}\right)+v\right).$$

For patient ASC23, tumour purity was estimated based on proportionate deleterious *PIK3CA* variant VAF in cfDNA and sphDNA compared to FFPE, made relative to FFPE estimated tumour purity (based on *TP53*). This sample was excluded from clonal analysis to avoid errors from miscalculated purity.

#### 2.4.3 Genomic alterations

Copy number profiles were estimated using CNVKit [29] with Katana [27]. CNVKit was calibrated with the TSO500 probe BED file, identifying 30 624 sites with high on‐ and off‐target coverage. Profiles were assessed against a flat reference and output was rescaled based on estimated tumour purity. The log_2_ copy ratio of read depth at each site was used to estimate local copy number. Segments of disparate copy numbers which were > 10 Mb in length, and adjacent to other > 10 Mb segments on the same chromosome, were considered large genome alterations (LGAs) [30]. Segments with read depth above a modified *Z*‐score of 3.5 were considered unreliable and assigned the average of the neighbouring segments' estimated copy number. Copy number profiles across the cohort were clustered using the ‘Clustermap’ function of Seaborn python package.

Loss of heterozygosity (LOH) was identified by counting the percentage of occupied chromosome‐limited 100 KB bins with average VAF deviating from 0.4 to 0.6. Where average read depth was below 150× (ASC017FFPE) or where median VAF was below 0.1 (ASC023FFPE), LOH% could not reliably be determined.

#### 2.4.4 Clonal analysis

Cancer clone clusters were estimated using PyClone‐VI [31]. VAF, read depth, estimated copy number, estimated tumour purity and expected normal copy (diploid) of potentially somatic variants were used as input. Beta‐binomial distribution and 10 random restarts were used. Variants with copy number of 0 in any matched sample cannot be analysed by the software. In one case (ASC28), the likely gatekeeping deleterious *TP53* variant could not be clustered for this reason, so a high frequency unrepresented clone was assumed, coloured grey. To limit clonal analysis to high confidence somatic mutations, where participants had any samples of < 30% tumour content, only variants of below 0.3 VAF in that sample were included in clonal analysis. Cancer cell frequency of clones at different time points was used to estimate possible phylogeny and evolution and representations were made using Adobe Illustrator.

#### 2.4.5 Oxford Nanopore Technology sequencing of sample ASC23

Reads per megabase (Mb) aligning to Chr17p13.1 (17:6 500 001–10 800 000) and 17q21.31 (42 800 001–46 800 000) were counted.

### 2.5 Statistics

Non‐parametric statistical tests, listed in associated figure legends, were performed using graphpad prism 9.5.1 software (GraphPad, Boston, MA, USA). Results were considered significant where *P*‐value was below 0.05.

## 3 Results

### 3.1 Cohort

This study recruited 15 participants, 14 with high‐grade serous tubal/ovarian/peritoneal cancer and one with clear cell ovarian cancer. Median age at recruitment was 61 years (age range, 36–82 years). Seven of the 15 participants were recruited with chemotherapy naïve disease, one after minimal prior chemotherapy, one at interval debulking surgery and six with recurrent disease (with all but one of these having had previous chemotherapy, Fig. 1B). Participant characteristics are listed in Table 1.

**Table 1:** Cohort details. cfDNA, ascites cell‐free DNA; dup., duplicate; FFPE, formalin‐fixed paraffin‐embedded tissue section DNA; HGS, high‐grade serous; n/a, not applicable; ser., serial; sphDNA, ascites spheroid cell DNA.

Research ID	Histotype	Age at recruitment	Chemotherapy history at recruitment	Tissue sequenced	Days between serial samples (cfDNA1 day 0)	Chemotherapy between cfDNA samples
*ASC6*	HGS	71	Naïve	cfDNA (1)	n/a	n/a
*ASC7*	HGS	78	Carboplatin	ser. cfDNA (2)	265	Paclitaxel, gemcitibine
*ASC9*	HGS	41	Naïve	ser. cfDNA (2)	319	Carboplatin, paclitaxel, pegylated liposomal doxorubicin, bevacizumab
				sphDNA (1)	0	
				FFPE (1)	831	
*ASC10*	HGS	82	Carboplatin, pegylated liposomal doxorubicin, tamoxifen	ser. cfDNA (2)	22	Nil
*ASC11*	HGS	58	Carboplatin, paclitaxel, veliparib/placebo, tamoxifen, pegylated liposomal doxorubicin, olaparib	ser. cfDNA (2)	36	Carboplatin, pegylated liposomal doxorubicin, paclitaxel
				sphDNA (1)	0	
*ASC12*	HGS	69	Naïve	ser. cfDNA (2)	13	Carboplatin, paclitaxel, durvalumab, tremelimumab
*ASC17*	HGS	80	Naïve	cfDNA (1)	n/a	n/a
				FFPE (1)	103	
*ASC20*	HGS	40	Carboplatin, paclitaxel, cisplatin, pegylated liposomal doxorubicin, olaparib, trabectidin	ser. cfDNA (2)	28	Cyclophosphamide
				sphDNA (1)	0	
*ASC23*	Clear cell	36	Naïve	ser. cfDNA (2)	559	Carboplatin, paclitaxel, bevacizumab, THOR‐707, pegylated liposomal doxorubicin, bevacizumab, olaparib, CYH33 (PIK3k inhibitor)
				sphDNA (1)	0	
				FFPE (1)	−287	
*ASC26*	HGS	71	Naïve	ser. cfDNA (2)	21	Carboplatin, paclitaxel
*ASC28*	HGS	58	Carboplatin, paclitaxel, bevacizumab, pegylated liposomal doxorubicin	ser. cfDNA (2)	78	Nil
				sphDNA (1)	0	
				FFPE (1)	−732	
*ASC30*	HGS	61	Carboplatin, paclitaxel, bevacizumab	dup. cfDNA (2)	0	n/a
				FFPE (1)	0	
*ASC36*	HGS	59	Naïve	ser. cfDNA (2)	17	Carboplatin, paclitaxel
*ASC37*	HGS	75	Carboplatin, paclitaxel, pegylated liposomal doxorubicin, olaparib cediranib/placebo, doxorubicin, bevacizumab	ser. cfDNA (2)	99	Carboplatin, gemcitabine
*ASC38*	HGS	49	Naive	cfDNA (1)	n/a	n/a

### 3.2 cfDNA from ascites is an exemplary template for next‐generation sequencing

#### 3.2.1 Alignment, coverage and tumour purity

Targeted sequencing was performed on cfDNA (*n* = 26), FFPE (*n* = 5) and sphDNA (*n* = 5) samples. No significant difference in genome alignment was observed (ordinary one‐way ANOVA, *P* = 0.61, Fig. 1C), but cfDNA achieved significantly higher median exon coverage (mean 915.9) compared to sphDNA (mean 610.8, *P* = 0.0057) and FFPE (332.6, *P* < 0.0001) by ordinary one‐way ANOVA and Dunnett's multiple comparisons test (Fig. 1D). One FFPE sample achieved MEC below the guideline of 150× (ASC17FFPE, MEC 105).

The tumour purity of cfDNA samples was estimated at 23–93%, with 14 of 15 cfDNA samples having > 30% tumour purity (Fig. 1E). By comparison, estimated tumour purity ranged from 10% to 90% in sphDNA, with 3 of 5 < 30% pure, and from 32% to 92% in FFPE. Comparing patient‐matched samples, cfDNA had higher purity than sphDNA in 3 of 5 cases (range 0.26–5.03×, median 2.93×) and higher purity than FFPE in 2 of 5 cases (range 0.29–1.22×, median 0.94×, Fig. S2A).

The TMB of cfDNA (range 1.6–9.4, median 4.7) was not significantly different to FFPE (Dunnett's multiple comparison's test, *P* = 0.10) or sphDNA (*P* > 0.99, Fig. 1D). Where comparing patient‐matched samples, cfDNA identified a median of 2.96× (range 1–6.88×) the TMB of sphDNA, and 1× (range 0.49–1.41×) the TMB of FFPE (considering only the three samples where artefacts were not detected, Fig. S2B).

#### 3.2.2 Identification of key clinical variants

**Fig. 2:**
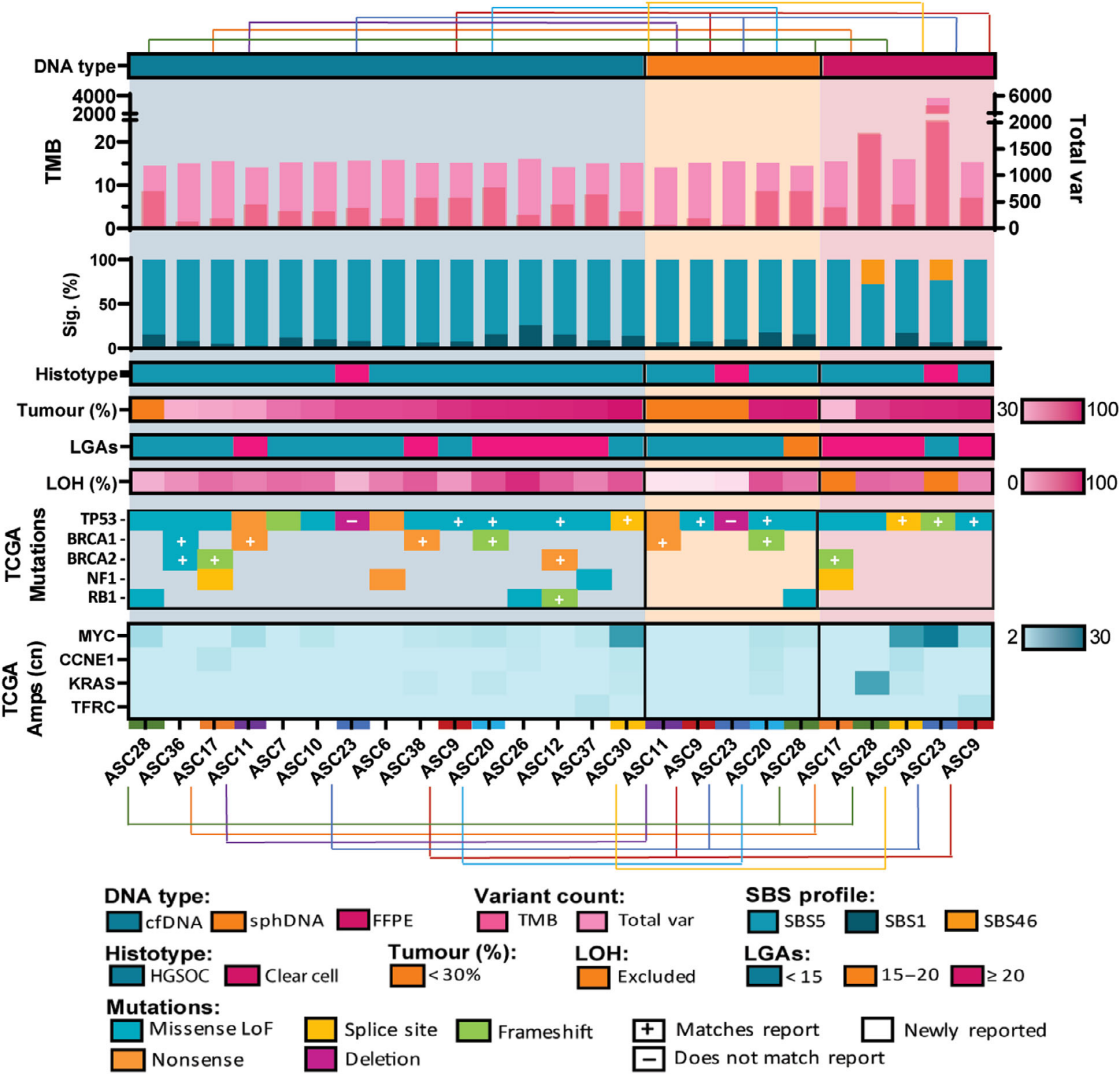
Sample characteristics, variant detection and genomic instability across cohort and sample types. cfDNA—cell‐free DNA; sphDNA—DNA from ascites‐derived cell spheroids; FFPE – DNA from formaldehyde‐fixed paraffin‐embedded tumour samples; TMB—tumour mutation burden, somatic variants per megabase (Mb), overlayed over total small variants (total var) detected (on right *Y*‐axis); Sig (%)—proportion of variants assigned to COSMIC single base substitution (SBS) signatures; Histotype—high‐grade serous (HGSOC) or clear cell histological subtype; Tumour (%)—estimated tumour proportion; LGAs—large‐scale genomic alterations, count of > 10 Mb segments with altered copy number to adjacent > 10 Mb segments; LOH (%)—loss of heterozygosity as a percentage of 100 kb bins where average non‐homozygous variant allele frequency is < 0.4 or > 0.6; Mutations—deleterious variants in genes identified by the Cancer Genome Atlas (TCGA) to be frequently mutated in ovarian cancer. ‘Matches report’ variants have been previously identified by clinical testing, ‘does not match report’ denotes conflicting variant to clinical testing, ‘newly reported’ variants are identified for the first time; LoF—loss of function; Amplifications—estimated copy number (cn) in genes identified by TCGA to be frequently amplified in ovarian cancer.

Somatic cancer driving variants were detected in all cfDNA samples (3–8 deleterious/VUS variants per patient) at VAFs of up to 0.88. Across the cohort, commonly altered genes included *TP53* (14 of 15), *BRCA1* (4 of 15, including 3 germline), *BRCA2* (3 of 15, including 1 germline), *NF1* (3 of 15), *RB1* (3 of 15) and *NOTCH1* (3 of 15) (Fig. 2, Fig. S3). Of these, all but *NOTCH1* are among the top 9 significantly recurrent genes identified in the TCGA ovarian carcinoma cohort [32].

Where results of clinical testing were available (11 of 15 cases), concordance was seen in cfDNA for 18 of 19 clinically reported variants (Table S1). The single mutation which was not identified in cfDNA was accounted for by a gene deletion event identified by CNVkit, discussed further in Section 3.3.2.

Gene amplifications were identified in 13 of 15 cfDNA samples (Fig. 2). The most commonly amplified genes included *MYC* (*n* = 9) and *PIK3CA* (*n* = 6), with other amplifications of potential clinical significance in *CCNE1* (*n* = 3) and *EGFR* (*n* = 2).

**Fig. 3:**
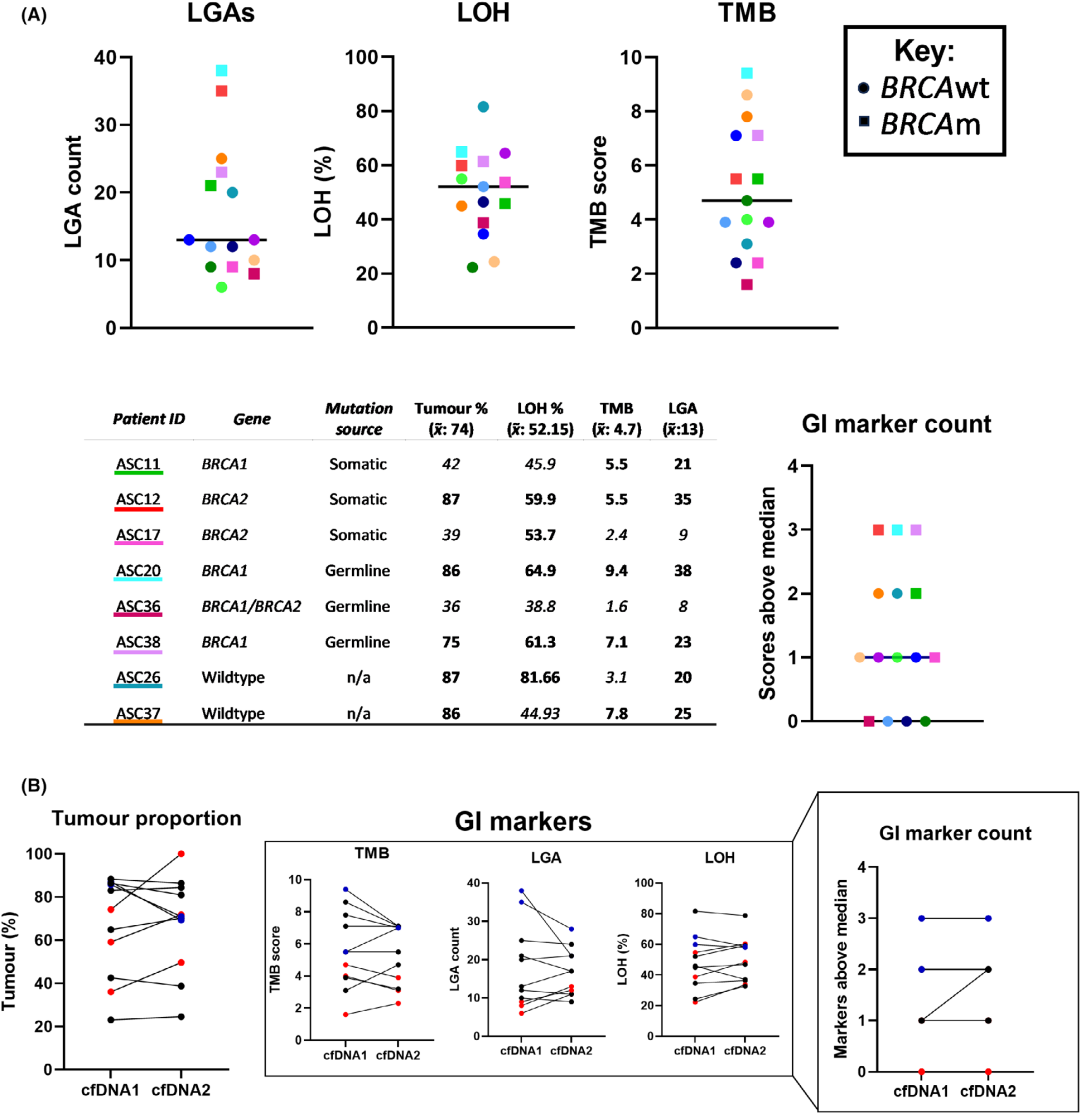
Consensus of genome instability markers in cell‐free DNA. (A) Number of large‐scale genomic alterations (LGAs), percent loss of heterozygosity (LOH) and tumour mutation burden (TMB) in initial cell‐free DNA samples are considered linked to genome instability and are indicated. Scores of individuals with known BRCA1/2 mutations or high consensus in genome instability are tabulated. Number of scores above the indicated median contribute to the genome instability (GI) marker count. Colours are assigned to individuals and maintained across charts. In table, all scores above median are in bold. (B) Markers of genomic instability in serial ascites samples, with sample pairs that varied in tumour proportion by more than 10% marked in red (increase) or blue (decrease) according to left‐most figure. Right‐most figure indicates number of markers above median for each individual. cfDNA, cell‐free DNA; m, mutant; wt, wild‐type; $\bar{x}$, median.

LOH, LGA and TMB were used as surrogate markers for genomic instability (Fig. 3A), samples were given genomic instability scores of 0–3 reflecting the number of markers with levels above the median. The three samples with genomic instability scores of 3 had *BRCA* mutations. Two additional *BRCA* wild‐type samples (with no other pathogenic variants in known HRD‐related genes) also scored highly, potentially harbouring *BRCA1* promoter methylation or another cause of HRD. Two *BRCA* mutant samples did not receive high genomic instability scores, though these samples had lower tumour purity (36–39% vs. 75–87%). All ascites samples had LOH in > 16% of the assessed genome, the HRD cut‐off used by Coleman et al., though this is likely because targeted, as opposed to whole genome sequencing was used [33].

When examining genome instability markers in sequential ascites samples (Fig. 3B) agreement between genomic instability score remained consistent in all but one instance (ASC9, due to an increase in LGA). However, the heightened LGA (17 vs 13) did not cross the threshold of 20 described by Eeckhoutte et al. [30] to indicate HRD. Other changes in genomic instability markers were inconsistent across markers and were largely associated with changes in tumour content, particularly for LGAs (Fig. 3B). This argues for the value of examining multiple genomic instability markers to make an assessment of HRD.

#### 3.2.3 Variant reporting accuracy and concordance across biospecimens

All cfDNA and sphDNA samples aligned to varying proportions of SBS1 and SBS5, both associated with ovarian cancer (Fig. 2, Fig. S4). The two FFPE samples with outlying number of variants had > 20% alignment to artefactual signature SBS46, indicative of possible sequencing artefact. These two samples also had the highest reported TMB and an outlying number of total variants, hindering confident identification of real mutations.

Identified deleterious variants were concordant across most patient‐matched samples, despite the fact that FFPE was collected at a different timepoint in most cases (Fig. S3). However, up to 7 unique variants were identified in cfDNA samples (including driver mutations in *KIT* and *NOTCH1*, 0.03 and 0.05VAF respectively, Fig. S5). Of the two participants in whom there was sufficient quality to verify variants in FFPE, one had 90% consensus (54 of 60) across all samples but the other had 10 (of 52) variants unique to FFPE. Among these, 8 were within a 3.2 kb span on 6q22.1 (within gene *ROS1*), with an average VAF of 0.06. The unique variants were identified to belong to a single clone, isolated to the FFPE sample.

In other cases, sample sets had similar clonal representation. However, unique clones were reported in ascites samples in three cases (2 in cell‐free DNA and 1 in sphDNA). Unique clones were also identified in FFPE in five participants (including 2 without confounding circumstances: no artefactual variants and adequate read depth over all samples).

Copy number variations were identified in all cfDNA samples. In a heatmap with unsupervised clustering of genome wide copy number profile (Fig. S6), patient‐matched sequential cfDNA and sphDNA samples successfully clustered together in all but one case. Contrastingly, 3 of 5 FFPE samples were incorrectly separated from their patient‐matched samples.

In a case study comparing duplicate cfDNA sequencing reports from a single ascites sample, tumour fraction, copy number segmentation and LOH all varied by only 3% (93.48% vs 96.28%, 74 vs 76 and 64.46% vs 62.28%, respectively), LGA varied by 1 (13 vs 14) and TMB was identical (3.9).

### 3.3 Temporal change is evident with opportunistic ascites cfDNA sampling

#### 3.3.1 Newly diagnosed versus recurrent disease

To investigate a temporal effect in participants recruited at different stages of a typical ovarian cancer disease trajectory, we compared genome instability markers in initial cfDNA samples from participants recruited before and after chemotherapy exposure.

We found no significant change in participants between chemotherapy status (Fig. S7), though a trend towards increased TMB was observed with chemotherapy exposure.

In the six participants with *BRCA1/BRCA2* mutations, we saw no evidence of reversion mutations in the mutated gene.

#### 3.3.2 Change between sequential ascites samples

Sequential ascites samples were collected from 11 participants, with intervals ranging from 13 to 559 days (average 132.5). We compared the paired cfDNA samples to consider both reproducibility and temporal changes.

**Fig. 4:**
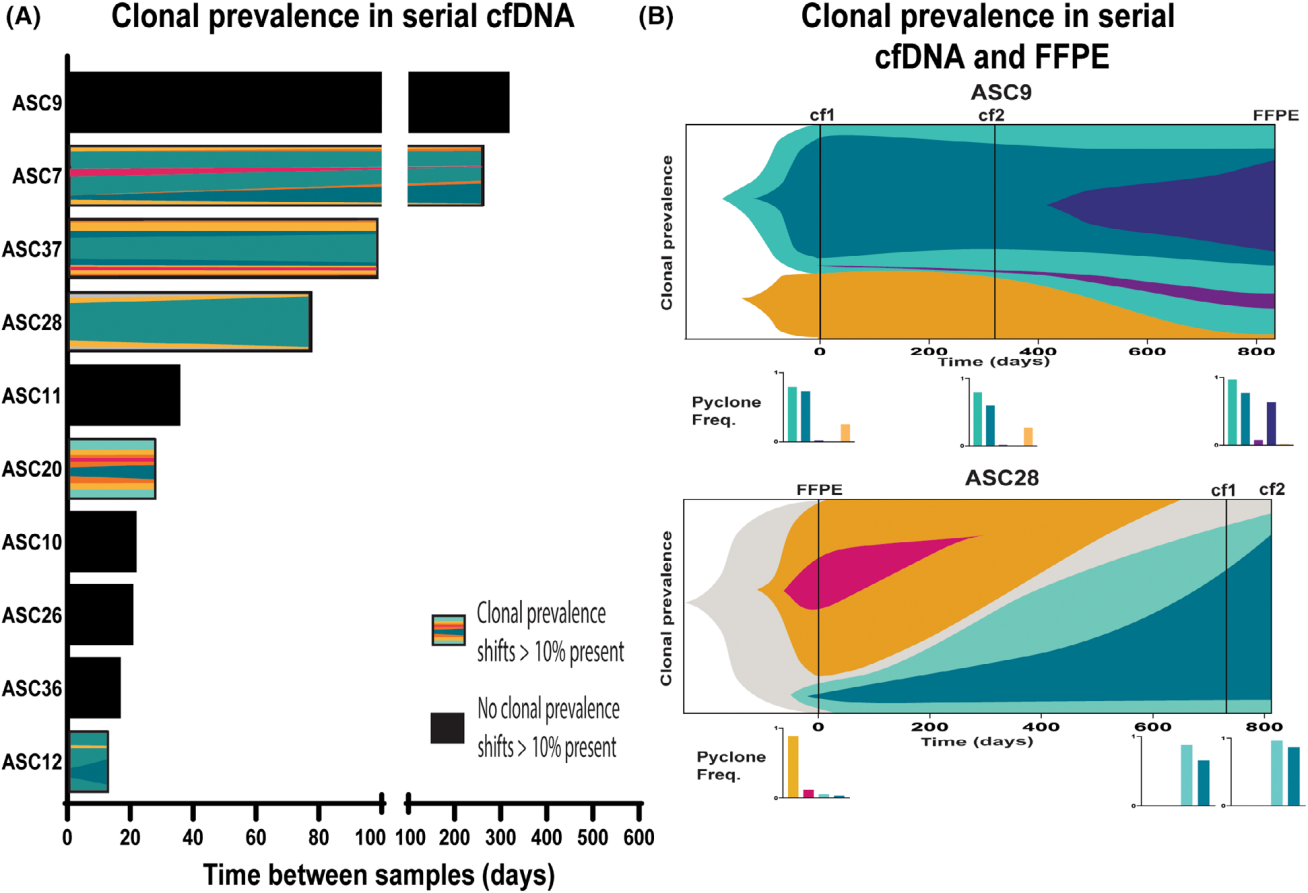
Temporal change in ascites cfDNA. (A) Representations of clonal prevalence in serial ascites samples where there are clone fraction changes > 10% between samples. (B) Representations of estimated clonal prevalence evolution in two samples based on clone frequencies estimated by PyClone‐VI at each of three timepoints. Colours represent independent clones identified by PyClone‐VI. cfDNA, cell‐free DNA; cf1/2, cell‐free DNA sample 1/2; FFPE, DNA from formaldehyde‐fixed paraffin‐embedded tumour samples.

We observed changes in copy number profile over time (Fig. S8), with disparity in 6.78–48.75% of sites (median 17.08). Though not significant, participants with more extensive chemotherapy history between samples tended to have larger divergence between samples (note participant ASC28 had tumour content below the recommended cut‐off of 30% for the copy number profiling, likely contributing to overestimation of copy number variation). Among clones identified in each participant, we identified five cases where clonal prevalence in at least one cluster altered by > 10% (Fig. 4A). No sample pairs had clones unique to one sample.

Among our cohort, three participants had samples collected at three timepoints (2× cfDNA and 1× FFPE), each with > 800 days between the first and last sampling. We observed differences in clonal prevalence in these samples with different time points and collection approaches (Fig. 4B). As artefactual variants were reported in ASC28FFPE, the validity of the uniquely reported clones in that sample is unclear.

In this small cohort, we did not observe any instances of driver mutation emergence or disappearance between cfDNA pairs. We also did not observe any instances of unique gene amplifications (> 2‐fold). We did not identify any significant changes in genome instability markers associated with amount of time/chemotherapy between samples (Fig. S9).

#### 3.3.3 Opportunistic liquid biopsy versus excisional tissue biopsy

**Fig. 5:**
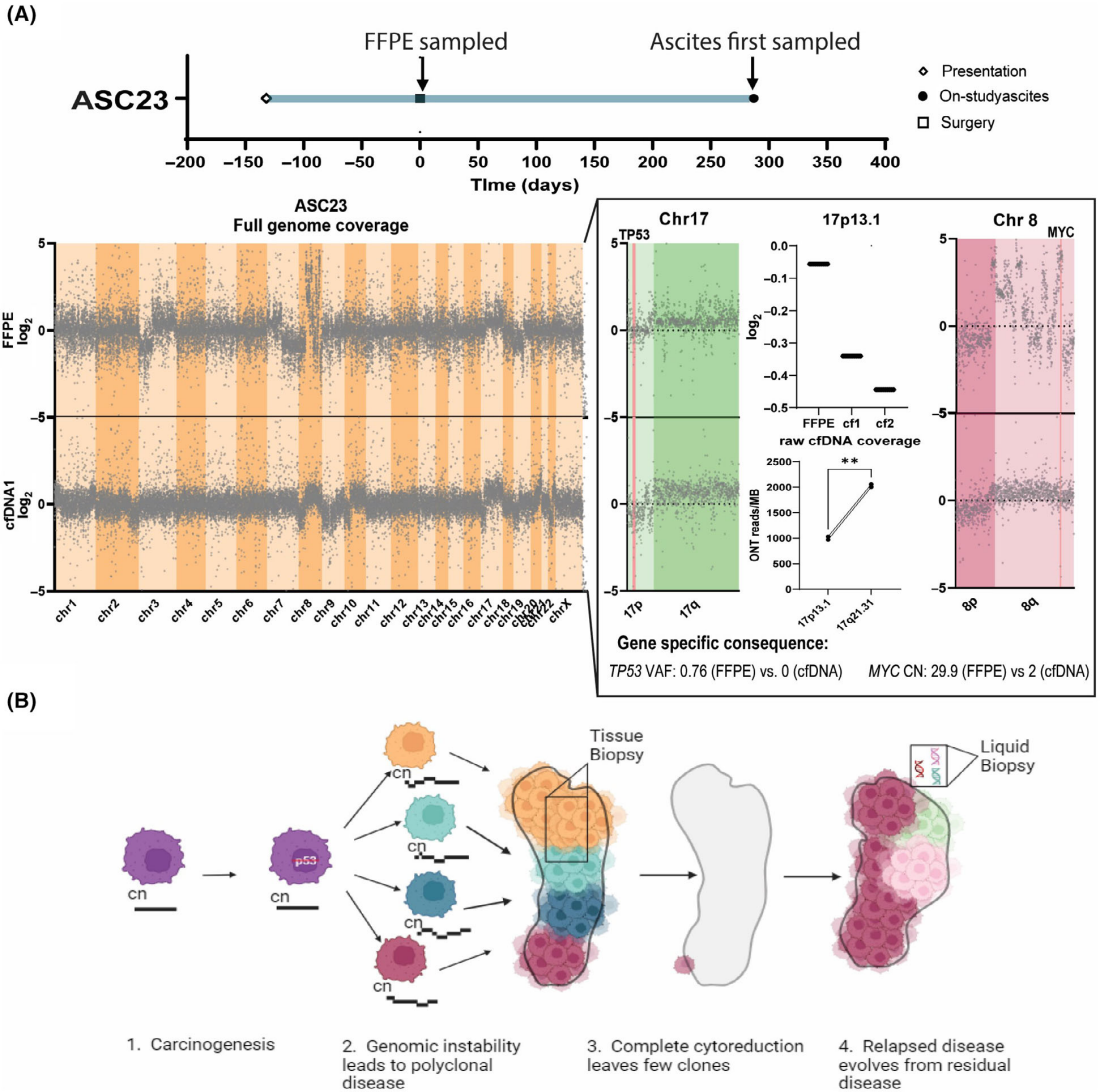
A case study of implications of tumour evolution with different spatial and temporal sampling. (A) Log_2_ read depth in cfDNA1 and FFPE samples collected from participant ASC23 approximately 10 months apart, with higher resolution reproductions of Chr17 and Chr8. Disparities in Chr17 are additionally represented by log_2_ of all variants on Chr17p13.1 in FFPE and serial ascites samples. Deletion of 17p13.1 is verified by paired *t*‐test of ONT reads per megabase (MB) on 17p13.1 and 17q21.31 in serial cfDNA samples (raw cfDNA coverage, *P* = 0.003). (B) Hypothesised mechanism explaining disparities between FFPE and cfDNA, owing to sampling technique and tumour evolution. Figure shows the development of a heterogeneous cancer, where the tissue biopsy taken at cytoreductive surgery is not representative of the residual clones that form relapse disease lesions and continue evolution after surgery. In this example, orange clones may have 1n copies of 17p13.1 (as seen in FFPE), whereas maroon clones may have 0*n* copies (as seen in cfDNA). cf1/2, cell‐free DNA sample 1/2; cfDNA, cell‐free DNA; Chr, chromosome; CN, copy number; FFPE, formalin‐fixed paraffin‐embedded; ONT, native Oxford Nanopore Technology sequencing; ONT, Oxford Nanopore Technology sequencing; VAF, variant allele frequency; **P < 0.01.

Though we were unable to reliably track clones in participant ASC23, due to a larger number of confounding artefactual variants in the FFPE, we did note some key differences between samples (Fig. 5A). Specifically, copy number profiling revealed discordance between FFPE and ascites samples, explaining previously noted differences in *TP53* mutation and *MYC* amplification (Fig. 2, Fig. S3). Notably, chr17p13.1 features a low log_2_ (−0.056) in the FFPE (consistent with the double‐hit hypothesis [28]), but significantly lower log_2_ (−0.34 and −0.44) in the ascites samples collected approximately 10 and 28 months later, respectively, suggesting a clonal loss of the second 17p13.1, along with the *TP53* mutation. This was confirmed with a decrease of alignment to 17p13.1 in cfDNA samples, relative to similarly sized and covered 17q21.31, by native whole genome nanopore sequencing (paired *t*‐test, *P* = 0.003). Chromosome 8q demonstrates severe instability in the FFPE sample, including a *MYC* amplification of 13× (copy number > 29), indicative of chromothripsis, which is not maintained in ascites samples. Theses disparities suggest that the clones captured in the FFPE were less prevalent when ascites was collected than would be indicated by analysing the archived FFPE biopsy, likely due to a combination of tumour evolution and sampling bias (Fig. 5B).

## 4 Discussion

cfDNA in ascites offers a clear opportunity for representative and not‐additionally invasive profiling of the ovarian cancer genome, enabling facilitation of precision medicine where tissue biopsies are contraindicated or compromised. This study has examined the feasibility, reliability and reproducibility of targeted sequencing output from ascites cfDNA and has captured unique evidence of tumour evolution identifiable with longitudinal sampling.

This study demonstrates that quality, representative sequencing data can be attained from ascites cfDNA. cfDNA was observed to be high in tumour content (up to 95%) with mutation profiles concordant with solid tumours, demonstrated by comparison to archived FFPE tissue, clinical reports and the Cancer Genome Atlas [32]. Mutations of key clinical relevance included three somatic *BRCA1/2* cases identifiable in cfDNA. Importantly, HRD was assessable in cfDNA. HRD samples with sufficient tumour content could be identified by LOH, LGAs and high TMB, extending on methods previously used for HRD assessment in cfDNA [15, 16]. Kfoury et al. and Roussel‐Simonin et al. recently reported a strong correlation in HRD scores between cfDNA and tissue biopsies [15, 16].

Among our cohort, ascites sampling preceded surgery in 7 of 15 participants, in one case by over 2 years. This was particularly key in 2 of the 3 cases with somatic *BRCA1/2* mutations, where ascites preceded surgery by 100 days or more. As sequencing turnaround time can be substantial [15], earlier knowledge of *BRCA1/2* mutations and HRD may be instrumental in the streamline delivery of maintenance PARPi or application of neoadjuvant PARPi, which is currently under investigation for efficacy [34]. Core biopsies have a > 25% failure rate for provision of adequate quality sequencing template [35], so ascites cfDNA may offer the most robust and timely alternative in the frontline setting.

In 9 of the 15 cases studied (to date), ascites emerged with disease relapse, where clinical trials of targeted therapies are often considered and archived tissue is accessed for biomarker profiling [36]. We have demonstrated the value of opportunistic ascites sampling by showing evidence of tumour evolution identifiable in sequential samples. Genome instability markers and copy number profiles were seen to change over time, in line with previous reports [22]. Furthermore, > 10% shifts in clonal prevalence were observed in 5 of 10 sequential ascites sample pairs. This, along with our key finding of a high prevalence of sequencing artefacts in 2 of 5 archived FFPE samples, demonstrates the improved likelihood of identifying and ascertaining the relevance of clinically actionable variants in ascites over archived FFPE. Although we did not observe driver mutation or *BRCA* reversion mutation emergence between cfDNA samples in this small cohort, this should be monitored again in a larger study.

One case showed particularly prevalent divergence between samples. When comparing FFPE from primary debulking surgery and cfDNA at disease relapse, deletion of both Chr17p13.1 and a chromothripsis‐affected Chr8q was observed, along with other significant copy number changes. These changes suggest active clonal selection throughout the course of the disease. As well as evolution, the limitation of sampling bias that is inherent to tissue biopsies may have played a part [37]. Ascites cfDNA, which flows freely through the peritoneal cavity, and is sampled in an unbiased manner, overcomes this limitation [37]. cfDNA is likely proportionally representative of peritoneal lesions, capturing intra‐tumour heterogeneity of ovarian cancer, which is usually peritoneally confined [38].

A strength of the present study is our report of the technical performance of cfDNA, using a standard commercially available next‐generation sequencing platform. cfDNA achieved significantly improved coverage over sphDNA and FFPE, subsequently improving capacity for variant identification and verification. Additionally, when we performed copy number profiling on sequential cfDNA samples across the cohort, 10 of 11 sample pairs clustered within participants, demonstrating reproducibility, with a trend towards more divergence in samples with extensive intervening chemotherapy. sphDNA similarly clustered in participant groups, however 3 of 5 FFPE samples incorrectly clustered, potentially indicative of susceptibility to copy number estimation errors. In the case study we performed of duplicate cfDNA sequencing, consensus in variant detection and copy number profile was observed.

The main limitation of this work was the small cohort size, hampering our ability to observe statistically significant differences. A larger cohort, covering a broader range of ovarian cancer histological subtypes, would provide greater confidence in our findings. We were also unable to compare ascites with fresh tissue as in most cases ascites was collected at routine paracentesis. This limited our assessment of concordance with tumour tissue, however, published works have previously demonstrated high similarity between ascites cfDNA and time‐matched fresh tumour tissue [14, 15, 16]. As ascites is often a contraindication for surgery and the accompanying opportunity for excisional biopsy, we believe the comparison with archived tissue is a better imitation of the clinical setting. Additionally, although this work demonstrated successful application of several bioinformatic analyses often reserved for whole genome‐spanning data sets, we were unable to compute clinically‐analogous HRD scores in the absence of either whole genome sequencing data or matched normal tissue DNA.

Future studies with larger cohorts and more samples for spatial, temporal and genomic reference could provide further confidence in cfDNA analysis, particularly for HRD assessment. A recent report showcased the high spatial and temporal variability of HRD scores in high‐grade serous ovarian cancer samples [39]. Considering the unbiased sampling possible with cfDNA analysis, it should be investigated whether cfDNA‐based HRD analysis can encapsulate the most reflective HR status at key time points and across tumour sites, allowing better prediction of PARPi efficacy. Similar assessment should be performed on new molecular biomarkers for targeted treatments, as they continue to emerge.

This study demonstrates the potential benefit for opportunistic sequencing of cfDNA from ascites to guide personalised medicine for ovarian cancer. We show cfDNA to provide exemplary template for targeted sequencing; being high in tumour content, producing no sequencing artefact and identifying key disease markers which evolve over time. With these findings supporting several recent publications, we argue for the immediate implementation of ascites cfDNA sequencing to inform precision medicine in lieu of invasive tissue biopsies.

## 5 Conclusion

This research indicates the suitability of cfDNA from ovarian cancer ascites for somatic mutation profiling and genome instability inference, to facilitate precision medicine. We show cfDNA to reliably identify clinically actionable information at key timepoints, using an opportunistic, not‐additionally invasive surrogate biopsy approach. This is of particular relevance at initial diagnosis where ascites precedes interval debulking surgery, or at disease relapse as an alternative to profiling archived formalin‐fixed tissue. The evidence presented here aligns with various recent reports, highlighting the utility of ascites fluid for genomic profiling in the clinical setting.

## Conflict of interest

During the preparation of this work the authors used ChatGPT (OpenAI) to assist with some code design for data analysis. After using this tool/service, the authors reviewed and edited the content as needed and take full responsibility for the content of the publication. KW declares potential financial conflict of interest due to stock ownership in the following companies that are developing cell‐free DNA based clinical assays: Guardant Heath; Exact Sciences; EpiGenomics AG. No other authors have conflicts of interest to declare.

## Author contributions

BW, KW and CEF contributed to conceptualisation. BW, MD, KW and CEF contributed to formal analysis. BW, EP, JD, YCL, VA and RA contributed to investigation. JD, YCL, VA, RA and MD contributed to resources. BW, EP, MC and MD contributed to data curation. BW contributed to writing – original draft and visualisation. BW, EP, JD, MC, YCL, VA, RA, MD, KW and CEF contributed to writing – review and editing. MC, MD, KW and CEF contributed to supervision. CEF and MD contributed to funding acquisition.

### Peer review

The peer review history for this article is available at https://www.webofscience.com/api/gateway/wos/peer‐review/10.1002/1878‐0261.13710.

## Supporting information


**Fig. S1.** Cohort selection.
**Fig. S2.** Tumour purity and tumour mutation burden in matched samples.
**Fig. S3.** Variant allele frequency and consensus in matched samples.
**Fig. S4.** COSMIC Single Base Substitution Signatures assigned to samples.
**Fig. S5.** Variant and clonal concordance in matched samples.
**Fig. S6.** Copy number consensus between samples.
**Fig. S7.** Tumour mutation burden and genome instability markers before and after chemotherapy.
**Fig. S8.** Copy number profile of serial cfDNA samples.
**Fig. S9.** Tumour mutation burden and genome instability markers in sequential ascites samples.
**Table S1.** Allele Frequency of clinically reported variants.

## Data Availability

The data that support the findings of this study are available on request from the corresponding author. The data are not publicly available due to privacy or ethical restrictions.
