# Supplementary material for: Cell‐free DNA from ascites identifies clinically relevant variants and tumour evolution in patients with advanced ovarian cancer
Source: Mol Oncol. 2024 Aug 8;18(11):2668–83. doi: 10.1002/1878-0261.13710 (PMC11547227; doi:10.1002/1878-0261.13710)
Supplement: Supplementary file 1 — Fig. S1. Cohort selection. Fig. S2. Tumour purity and tumour mutation burden in matched samples. Fig. S3. Variant allele frequency and consensus in matched samples. Fig. S4. COSMIC Single Base Substitution Signatures assigned to samples. Fig. S5. Variant and clonal concordance in matched samples. Fig. S6. Copy number consensus between samples. Fig. S7. Tumour mutation burden and genome instability markers before and after chemotherapy. Fig. S8. Copy number profile of serial cfDNA samples. Fig. S9. Tumour mutation burden and genome instability markers in sequential ascites samples. Table S1. Allele Frequency of clinically reported variants. [file MOL2-18-2668-s001.docx]

***Supplementary Figure 1****. Cohort selection, based on meeting criteria in order of priority: recurrent ascites (Rec. Asc.), i.e. ascites collected at multiple timepoints, BRCA mutations in clinical report (BRCAm), time matched tissue sample from surgery available, non-BRCA somatic mutations in clinical report. 15-person final cohort above line.*


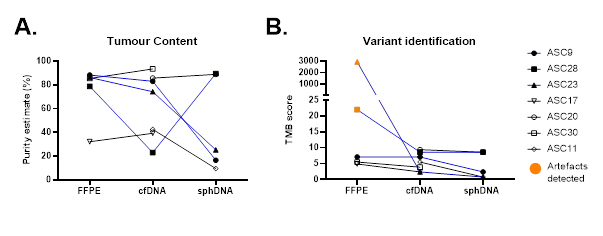


***Supplementary Figure 2.*** *Tumour purity (A) and TMB score (B) in cfDNA, sample-matched sphDNA and non-time-matched FFPE. Participants with full set available are identified by filled shapes and blue lines. Orange markers (B) indicate artefactual single base substitution signatures were identified.*


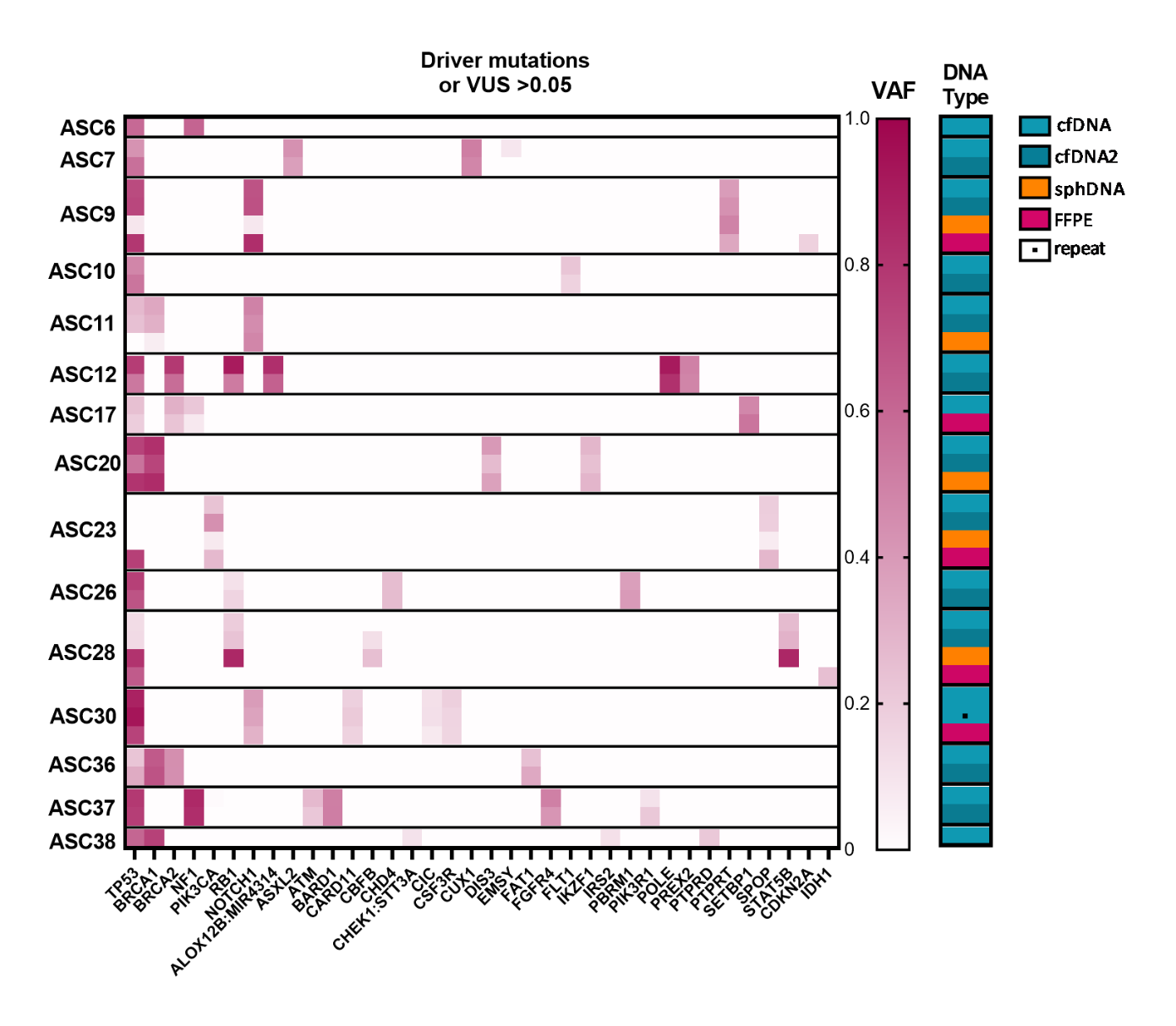
 ***Supplementary Figure 3.*** *Variant allele frequency and consensus between samples in cancer driving mutations with >5% VAF. cfDNA, cell-free DNA; sphDNA, DNA from ascites-derived cell spheroids; FFPE, archived tumour tissue.*

**Supplementary Table 1. Allele Frequency of clinically reported variants**

| ***Patient ID*** | ***gene*** | ***test*** | ***Clinically identified variant*** |  | ***VAF*** | | | |
| --- | --- | --- | --- | --- | --- | --- | --- | --- |
|  |  |  |  | ***variant type*** | ***cfDNA 1*** | ***cfDNA 2*** | ***sphDNA*** | ***FFPE*** |
| ASC6 | *TP53* | Somatic | p.C242Afs*5 | Frameshift | 0.59 | NT | NT | NT |
|  | *NF1* | Somatic | p.Y49* | Nonsense | 0.61 | NT | NT | NT |
| ASC9 | *TP53* | Somatic | p.R273C | Missense LOF | 0.71 | 0.73 | 0.09 | 0.79 |
| ASC11 | *BRCA1* | Somatic | p.K1606Lfs*18 | Frameshift | 0.34 | 0.31 | 0.06 | NT |
| ASC12 | *TP53* | Somatic | p.S241Ffs*23 | Frameshift | 0.77 | 0.53 | NT | NT |
|  | *BRCA2*  *RB1* | Somatic  Somatic | p.Y748*  p.L468* | Nonsense  Nonsense | 0.79  0.91 | 0.58  0.52 | NT  NT | NT  NT |
| ASC17 | *BRCA2* | Somatic | p.W2169* | Nonsense | 0.31 | NT | NT | 0.23 |
|  | *RAD51D* | Genomic | p.C9S (VUS) | Missense (Amb) | 0.57 | NT | NT | 0.59 |
| ASC20 | *BRCA1* | Genomic | p.L785* | Nonsense | 0.82 | 0.74 | 0.84 | NT |
|  | *TP53* | Somatic | p.P278A | Missense LOF | 0.75 | 0.55 | 0.80 | NT |
| ASC23 | *TP53* | Somatic | p.R196* | Nonsense | 0 | 0 | 0 | 0.76 |
|  | *PIK3CA* | Somatic | p.E545K | Missense GOF | 0.2324 | 0.4364 | 0.0792 | 0.27 |
| ASC28 | *PALB2* | Unknown | p.S1102R (VUS) | Missense LOF | 0.48 | 0.50 | 0.50 | 0.52 |
| ASC30 | *TP53* | Somatic | c.672+1G>T | Splice site | 0.88 | NT | NT | 0.74 |
| ASC36 | *BRCA1* | Genomic | p.V1736A | Missense LOF | 0.65 | 0.68 | NT | NT |
|  | *BRCA2* | Genomic | p.D3073G | Missense LOF | 0.44 | 0.44 | NT | NT |
| ASC38 | *BRCA1* | Genomic | p.L392GInfs*5 | Frameshift | 0.75 | NT | NT | NT |
|  | *TP53* | Somatic | p. Y236C | Missense | 0.61 | NT | NT | NT |

NT, not tested; LOF, loss of function; GOF, gain of function; Amb, ambiguous effect

**
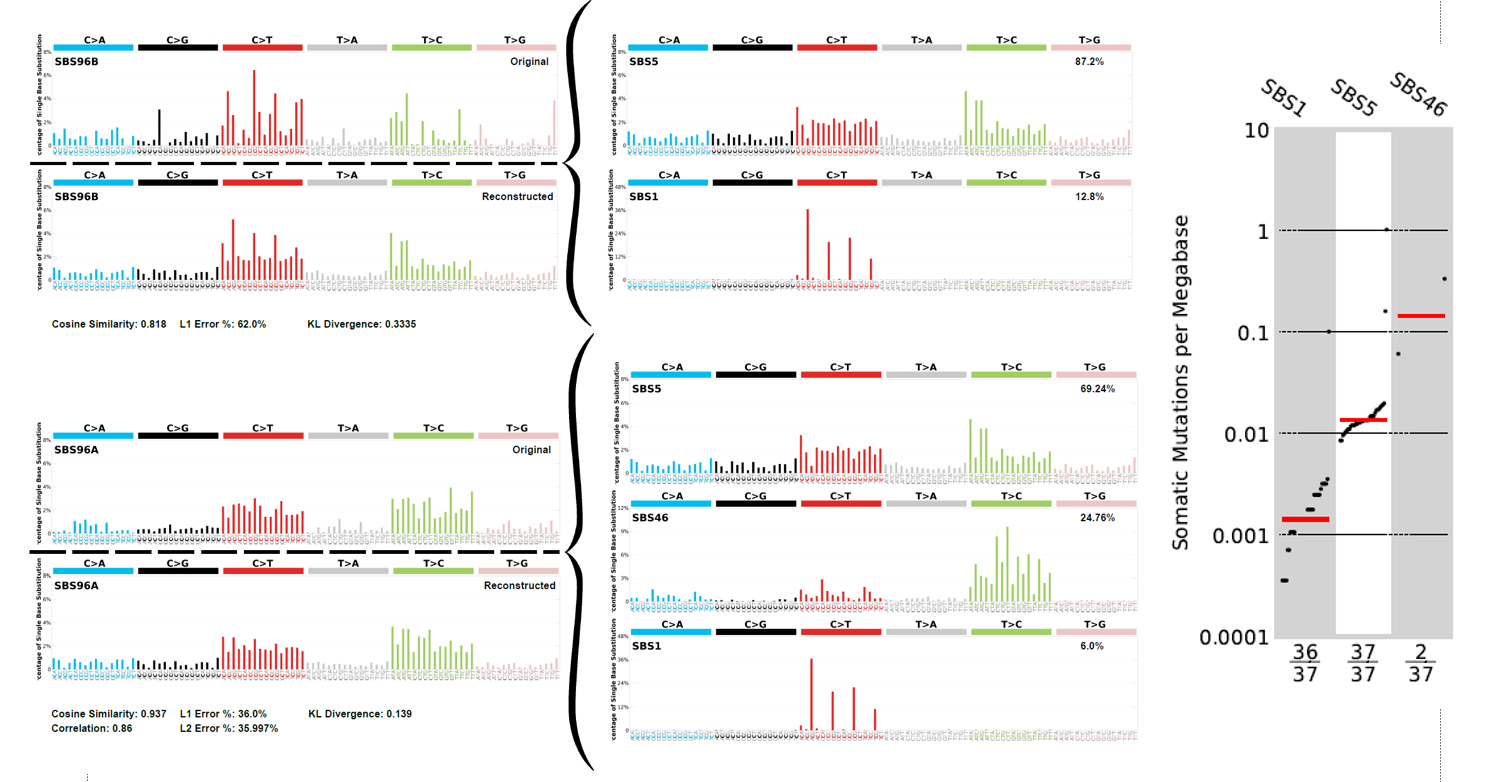
**

***Supplementary Figure 4.*** *COSMIC Single Base Substitution Signatures assigned to samples*


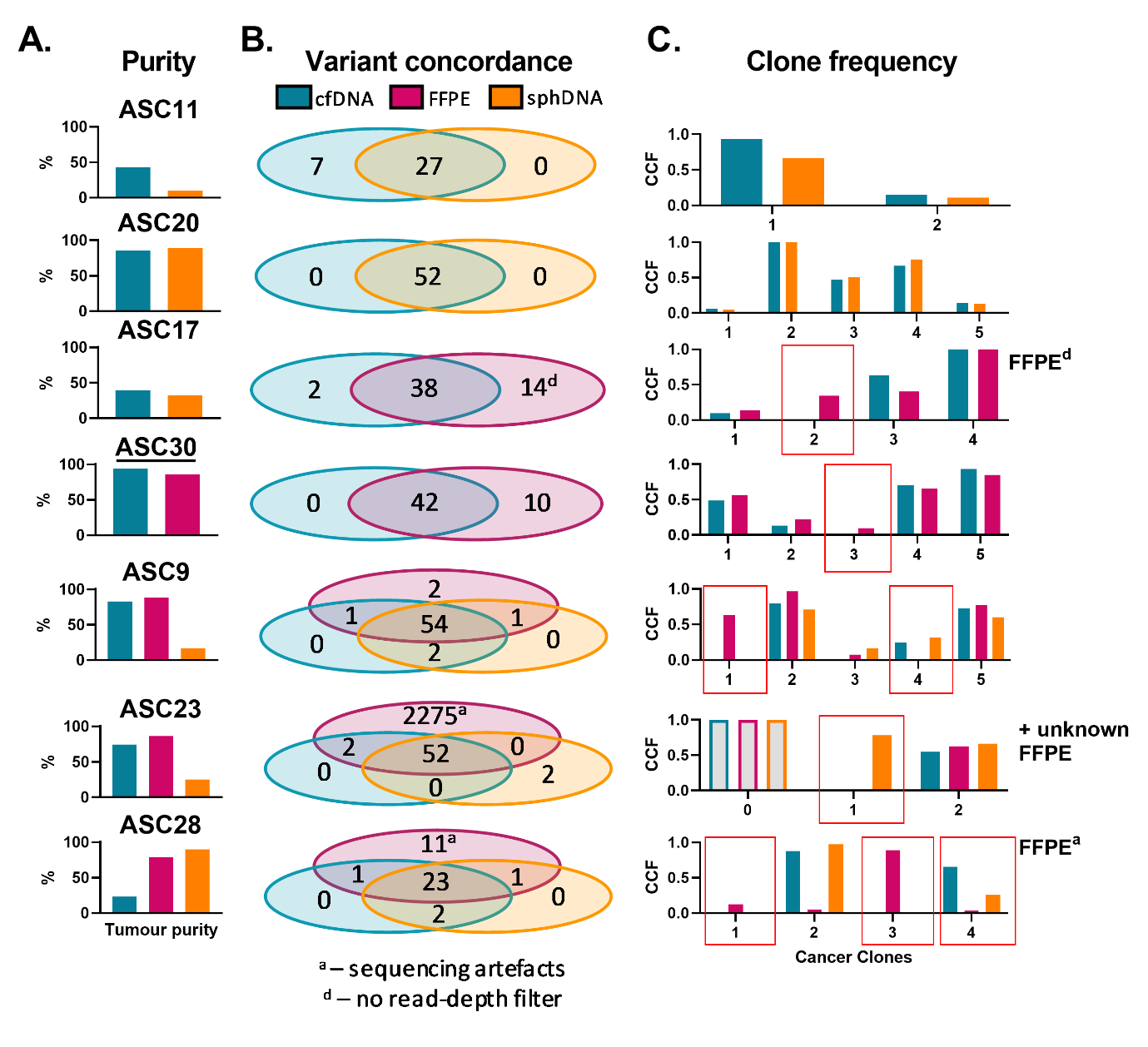


***Supplementary Figure 5.*** *Tumour purity estimate (A).* *Somatic variants (B). Cancer cell fraction (CCF) in clonal clusters identified by PyClone-VI (C). Red boxes outline clones not common across sample set. All cfDNA and sphDNA collected at matched timepoints. ASC30 (underlined) FFPE and cfDNA were collected at matched timepoint, all other FFPE was collected independently. CCF, cancer cell fraction; cfDNA, cell-free DNA (blue); sphDNA, ascites cell spheroid DNA (orange); FFPE, archived tumour tissue (maroon).*

**
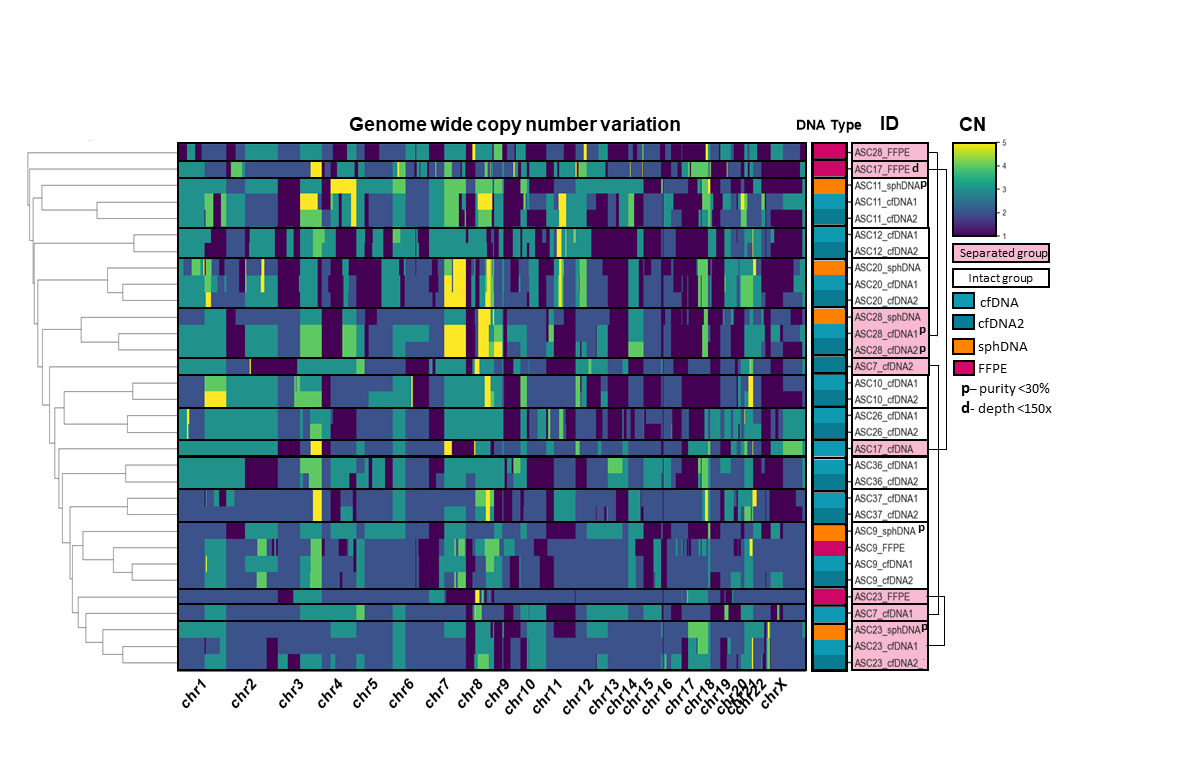
**

***Supplementary Figure 6.*** *Copy number consensus between samples.* Copy number profiles generated by CNVKit.


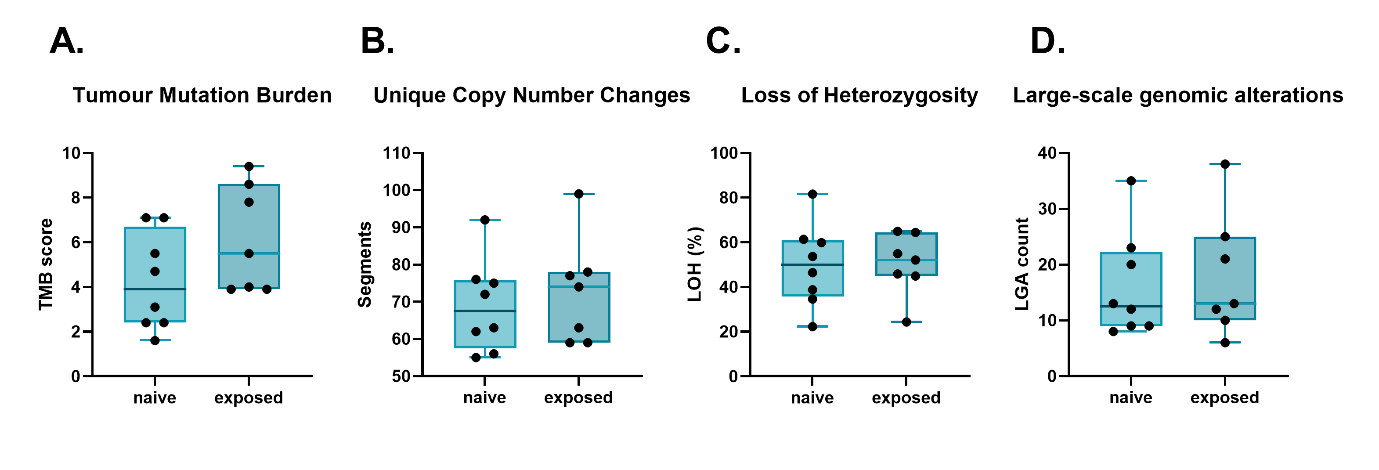


***Supplementary Figure 7.*** *Tumour mutation burden (TMB) (A), copy number segmentation (B) and loss of heterozygosity (C) in initial cell-free DNA samples, separated by preceding chemotherapy exposure, analysed by Mann-Whitney test (no significance).*


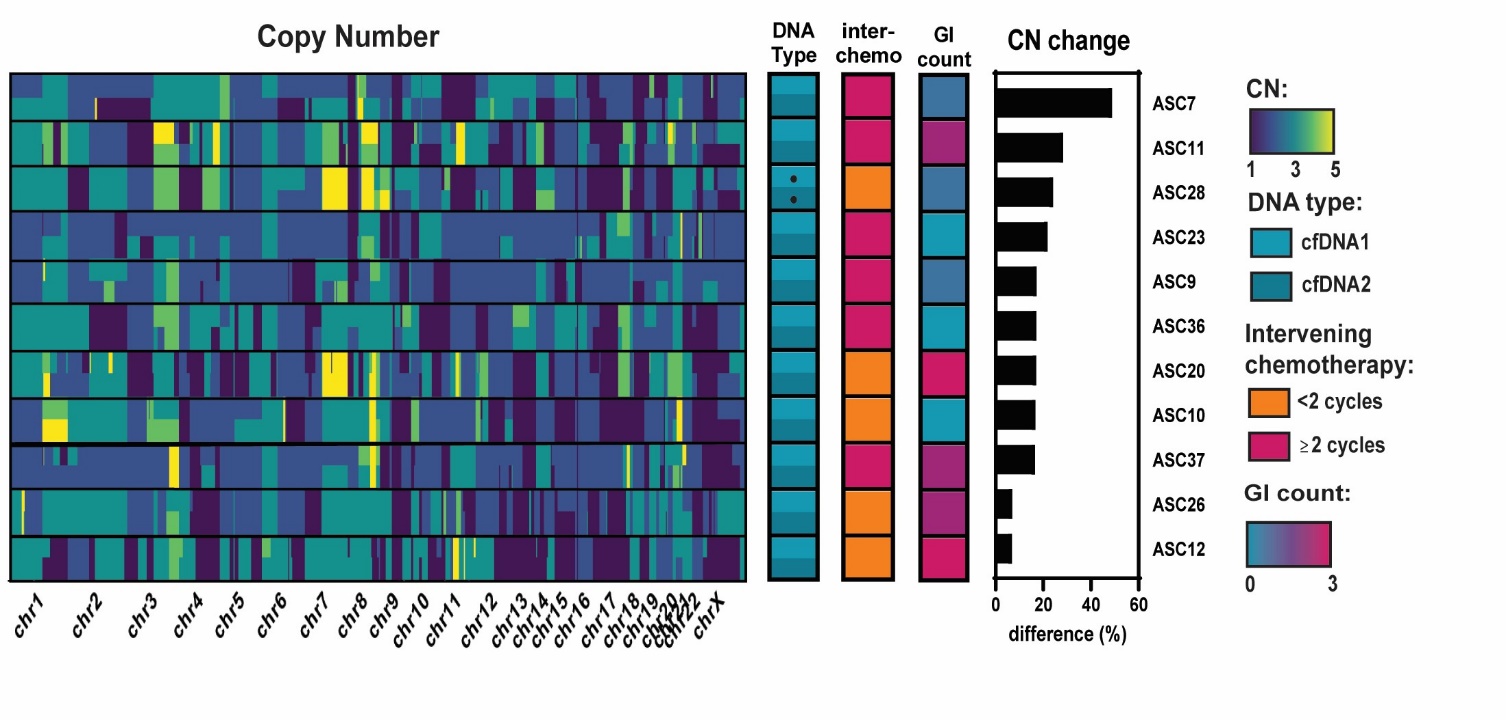


***Supplementary Figure 8.*** *Copy number profile of serial cfDNA samples, and calculated percentage of queried sites with disparate copy number profiles, in association with intervening chemotherapy history. cfDNA, cell-free DNA; CN, copy number; GI, genomic instability.*


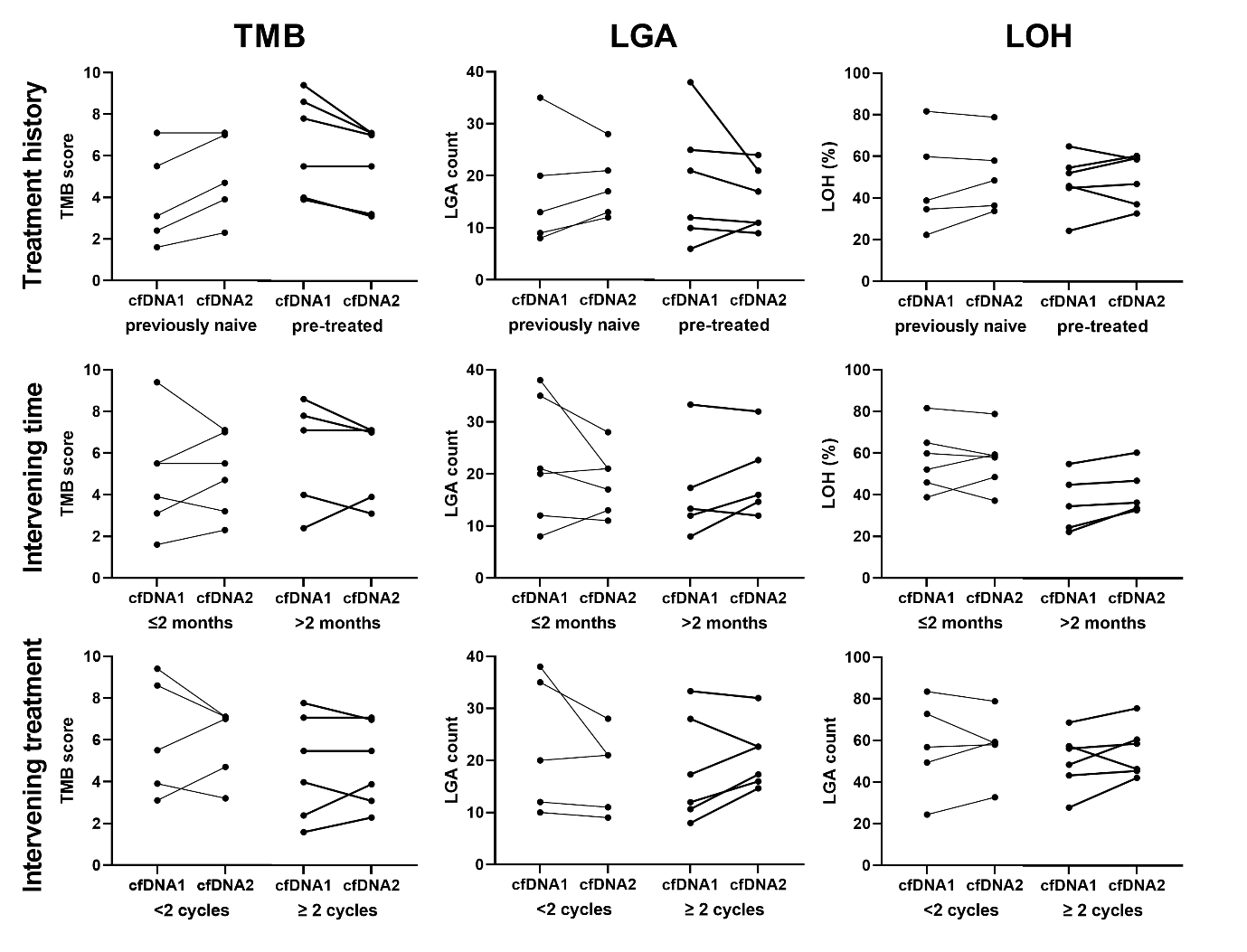


***Supplementary Figure 9.*** *Tumour mutation burden (TMB) large-scale genomic alterations (LGA) and loss of heterozygosity (LOH) in sequential ascites samples, separated into individuals with disparate treatment history, intervening time length and amount of intervening treatment.*
